# Towards better digital pathology workflows: programming libraries for high-speed sharpness assessment of Whole Slide Images

**DOI:** 10.1186/1746-1596-9-S1-S3

**Published:** 2014-12-19

**Authors:** David Ameisen, Christophe Deroulers, Valérie Perrier, Fatiha Bouhidel, Maxime Battistella, Luc Legrès, Anne Janin, Philippe Bertheau, Jean-Baptiste Yunès

**Affiliations:** 1 Laboratoire LIAFA - CNRS UMR 7089/Université Paris Diderot, Sorbonne Paris Cité, F-75205 Paris Cedex 13, France; 2 IMNC - UMR 8165 CNRS/Université Paris-Diderot, Université Paris-Sud, F-91405 Orsay, France; 3 Laboratoire Jean-Kunztmann, Université de Grenoble/CNRS, UMR 5224, 38041 Grenoble Cedex 9, France; 4 Laboratoire de Pathologie, Inserm UMR_S-1165/Université Paris-Diderot, Sorbonne Paris Cité, F-75010 Paris, France

## Abstract

**Background:**

Since microscopic slides can now be automatically digitized and integrated in the clinical workflow, quality assessment of Whole Slide Images (WSI) has become a crucial issue. We present a no-reference quality assessment method that has been thoroughly tested since 2010 and is under implementation in multiple sites, both public university-hospitals and private entities. It is part of the FlexMIm R&D project which aims to improve the global workflow of digital pathology. For these uses, we have developed two programming libraries, in Java and Python, which can be integrated in various types of WSI acquisition systems, viewers and image analysis tools.

**Methods:**

Development and testing have been carried out on a MacBook Pro i7 and on a bi-Xeon 2.7GHz server. Libraries implementing the blur assessment method have been developed in Java, Python, PHP5 and MySQL5. For web applications, JavaScript, Ajax, JSON and Sockets were also used, as well as the Google Maps API. Aperio SVS files were converted into the Google Maps format using VIPS and Openslide libraries.

**Results:**

We designed the Java library as a Service Provider Interface (SPI), extendable by third parties. Analysis is computed in real-time (3 billion pixels per minute). Tests were made on 5000 single images, 200 NDPI WSI, 100 Aperio SVS WSI converted to the Google Maps format.

**Conclusions:**

Applications based on our method and libraries can be used upstream, as calibration and quality control tool for the WSI acquisition systems, or as tools to reacquire tiles while the WSI is being scanned. They can also be used downstream to reacquire the complete slides that are below the quality threshold for surgical pathology analysis. WSI may also be displayed in a smarter way by sending and displaying the regions of highest quality before other regions. Such quality assessment scores could be integrated as WSI's metadata shared in clinical, research or teaching contexts, for a more efficient medical informatics workflow.

## Background

Since microscopic slides can now be automatically digitized and integrated in the clinical workflow, quality assessment of these Whole Slide Images (WSI) has become a crucial issue. Until now, the quality of a WSI has been verified a posteriori by a technician or by a pathologist. There is however a significant amount of WSI that are too insufficient in quality (blurred, bad colors, poor contrast) to be used for diagnoses. These slides have then to be scanned again with delay thus slowing down the diagnostic workflow. To address this problem, we chose to design a method of quality assessment followed by reacquisition, as opposed to a process of enhancement or restoration [1,2]. Such process indeed too frequently results in the degradation of image quality, a key factor in medical diagnosis. The quality of a flat image can be defined by several quantifiable parameters such as color, brightness, and contrast. One of the most important parameters, yet difficult to assess, is the focus sharpness (i.e. the level of focus blur) [3]). Quality assessment of WSI is much more complex than that of flat images because of their intrinsic structure made of multiple magnification levels (pyramidal structure) and resolutions above the gigapixel. One study [4] has shown the possibility of comparing the tiles' contrast and entropy in two WSI obtained with two different scanners digitizing the same slide. Another work [5] assessed the focus sharpness of the tiles of a WSI with the generation of a focus assessment map of the WSI at a given magnification level. However, both these methods still require a human eye to assess if the WSI must be accepted or discarded after the scan [6].The method we designed to automatically assess the quality of a WSI without any sort of comparison (no-reference assessment) has been patented [7] and thoroughly tested in the last four years. It is currently being implemented in our university-hospital Saint-Louis - Assistance Publique - Hôpitaux de Paris (APHP) - Université Paris Diderot - Paris 7, in Paris, France. It is also part of the FlexMIm project [8], which aims to improve the global workflow of digital pathology. This project, funded by an R&D grant of the French government for highly innovative technologies, also involves universities Paris 6 (LIP6 and IPAL laboratories) and Paris 7 (LIAFA laboratory) and industrial partners Orange Healthcare, Pertimm and TRIBVN as well as 27 anatomo-pathological centers in Paris and its suburbs. For these projects, we have developed two programming libraries, in Java and Python, that can be integrated in various types of WSI and image handling applications.

## Methods

The development has been carried out on a MacBook Pro Intel Core i7 2.6GHz, 16GB RAM, 512GB SSD, and the tests were carried out in University Paris Diderot Paris 7, with the following configuration: 2 Intel Xeon E5-2680 2.70GHz, 20M Cache, 8.0GT/s QPI, 24GB RDIMM, 1333MHz FBD RAM, 146GB SAS 6Gbps 15k RAID 1, 5 2TB SAS 6Gbps 7.2k RAID 5.

The tiles of each magnification level of the WSI need to be accessible to perform the analysis. Many open-source programs [9-11] as well as proprietary ones [12] can be used to extract WSI files from different formats (3dHistech, Aperio, Hamamatsu, Olympus) into series of tiles at different magnification levels.

Any WSI can be converted, at a given magnification level, into a series of tiles or strips (wider tiles) indexed by their (x,y) coordinates. Once the tiles of each magnification level are extracted, the saturation of each of them is computed. In every system, many "blank tiles" are stored because they contain visual artifacts detected as regions of interest but do not contain any specimen. As these blank tiles have saturation values close to zero, our system discards them from the set of images to analyze, saving from 5% (when the sample takes most of the WSI) to 90% (in blank WSI, containing no sample at all) of the time required to complete an analysis of a virtual slide at maximum magnification.

The remaining tiles are then analyzed with different tests such as blurriness, contrast, brightness and color. More tests can be integrated as plug-ins in the program. For the blurriness assessment we used our fast reference-free method designed to compute accurately the amount of blur in a single tile based on an edge brightness ratio [7]. Other tests such as contrast, brightness and color assessment are a result of computations made on the tile's pixels values, compared with their respective thresholds. For instance, one test could be to check if more than 90% of the pixels color values inside a tile were contained in three ranges of color.

Each tile receives quantitative and qualitative scores for each of the analyzed parameters and are compared to their respective thresholds. Note that the tiles can be virtually split to add granularity and refine the final assessment. For instance, at a 2× magnification, if more than 90% of the tiles are considered sharp, the complete 2× layer of the WSI is considered as sharp. If more than 70% of the 10× magnification is considered sharp, the 10× layer of the WSI is considered as sharp.

The analysis can be limited to the lower magnification levels of a WSI for a quicker result or extended to the highest magnification level for a more comprehensive quality assessment.

Once the tile analysis is done, if the WSI passed the quality assessment tests at each processed layer of magnification, the WSI is suitable for further use.

In order to test and validate the method, we analyzed a series of 100 WSI made of a mix of WSI with optimal focus and of WSI with various blurred areas, some of them being obviously totally blurred. We compared the computer assessment of these WSI to the human assessment in two settings:

- We first presented the 100 WSI in a random order to two observers from our research team.

- We then conducted a web survey [13] among 22 trained pathologists, asking them whether the overall quality of each WSI seemed sufficient for a clinical use. The human assessment was distributed among three possible answers: Poor; Fair; Good. The computer assessment represented the computed highest acceptable magnification for a WSI, higher magnifications being therefore considered by the computer as of insufficient quality for diagnosis.

The libraries implementing the blur assessment method we designed have been developed in Java, Python, PHP5 and MySQL5 using Eclipse IDE, Apache HTTP Server.

For web usage, JavaScript, Ajax, JSON and/or Sockets were used for multithreaded interactions between the web application hosted on one server, the java or Python services hosted on the same server, or a different (decentralized) one and the files stored on the same server or on a decentralized storage server.

We also used the Google Maps API, as demonstrated in the NYUVM (NYU's virtual microscope, developed by NYU school of medicine) [14]. Native reading of NDPI files was carried out using a modified version of Matthias Baldauf's NDPI to OME-TIFF Converter [15]. Aperio SVS files were converted into the Google Maps format using VIPS and Openslide libraries [16].

## Results and discussion

In the following, we use the blur assessment method described in the method section as an example to describe any other quantifiable criterion in an image, to be used a fortiori to assess the quality of WSI.

The complete quality assessment method is a logical intersection of independent tests, marking a WSI as of insufficient quality if at least one of the tests fails.

**Figure 1:**
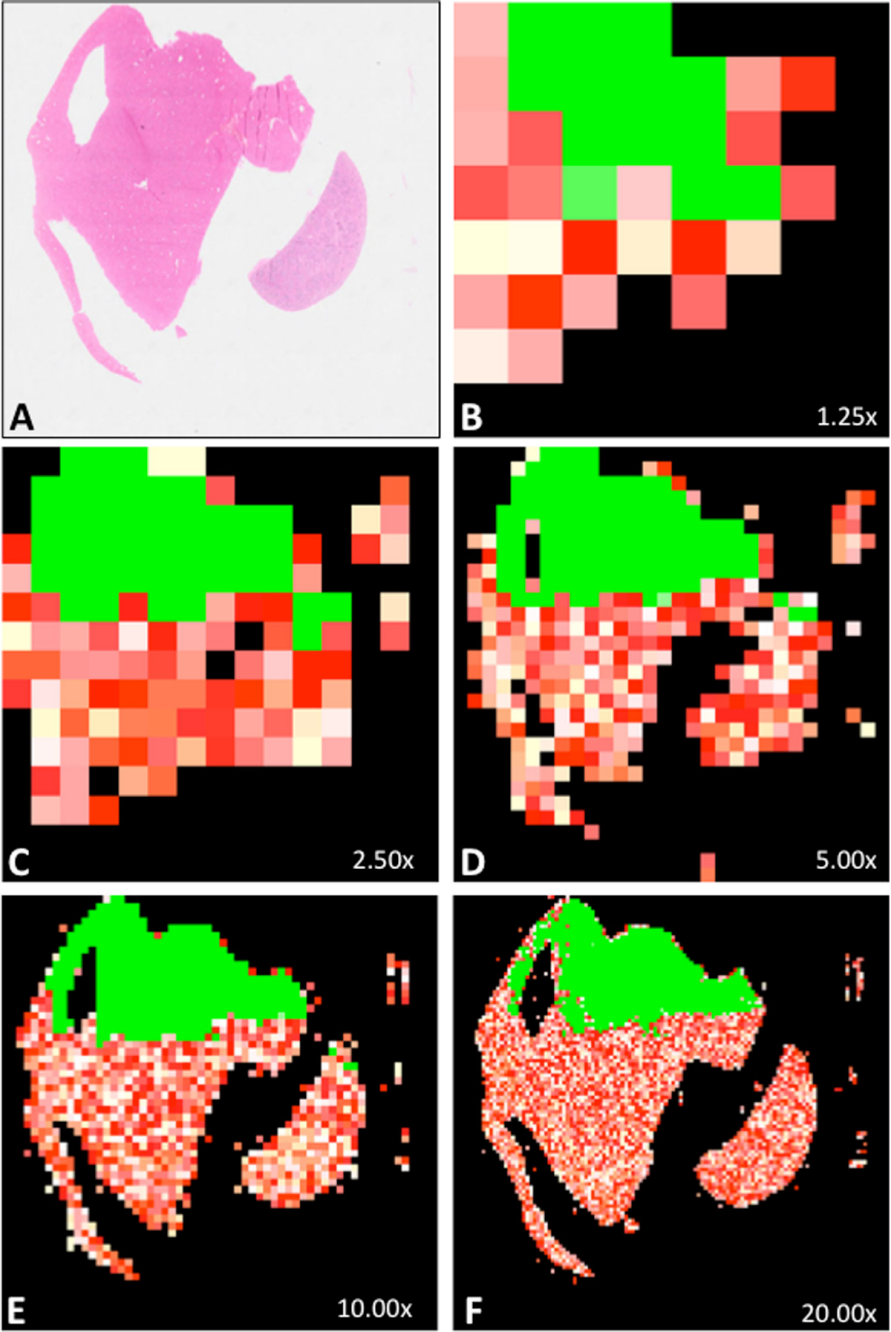
**Automatic quality analysis of a virtual slide (parameter used: blur)**. A represents the thumbnail of a whole slide image (H&E staining) whose upper third part is in focus and lower two thirds part is totally out of focus. Each thumbnail B to F shows sharp tiles in green and blurry tiles going from white (a little blurry) to red (the most blurry). Out of 43 tiles at 1.25× (B), 83% were detected as non-blank, and 36% were detected as sharp. For C, D, E and F, the respective values were (146 tiles, 2.5×, 86% non-blank, 34% sharp), (493 tiles, 5.0×, 83% non-blank, 33% sharp), (1751 tiles, 10.0×, 77% non-blank, 31% sharp), (6589 tiles, 20.0×, 76% non-blank, 25% sharp). The WSI is thus considered as of insufficient quality in terms of blurriness, for all its magnification levels being under their respective blur assessment thresholds.

We applied the quality analysis routine with the blur assessment parameter on hundreds of WSI. An example of automatic blur assessment is shown in Figure 1.

On a collection of 100 WSI, two observers could easily assess the overall level of quality they observed and they visually verified that the thresholds we set were highly predictive of the global sharpness or blurriness of the WSI.

**Figure 2:**
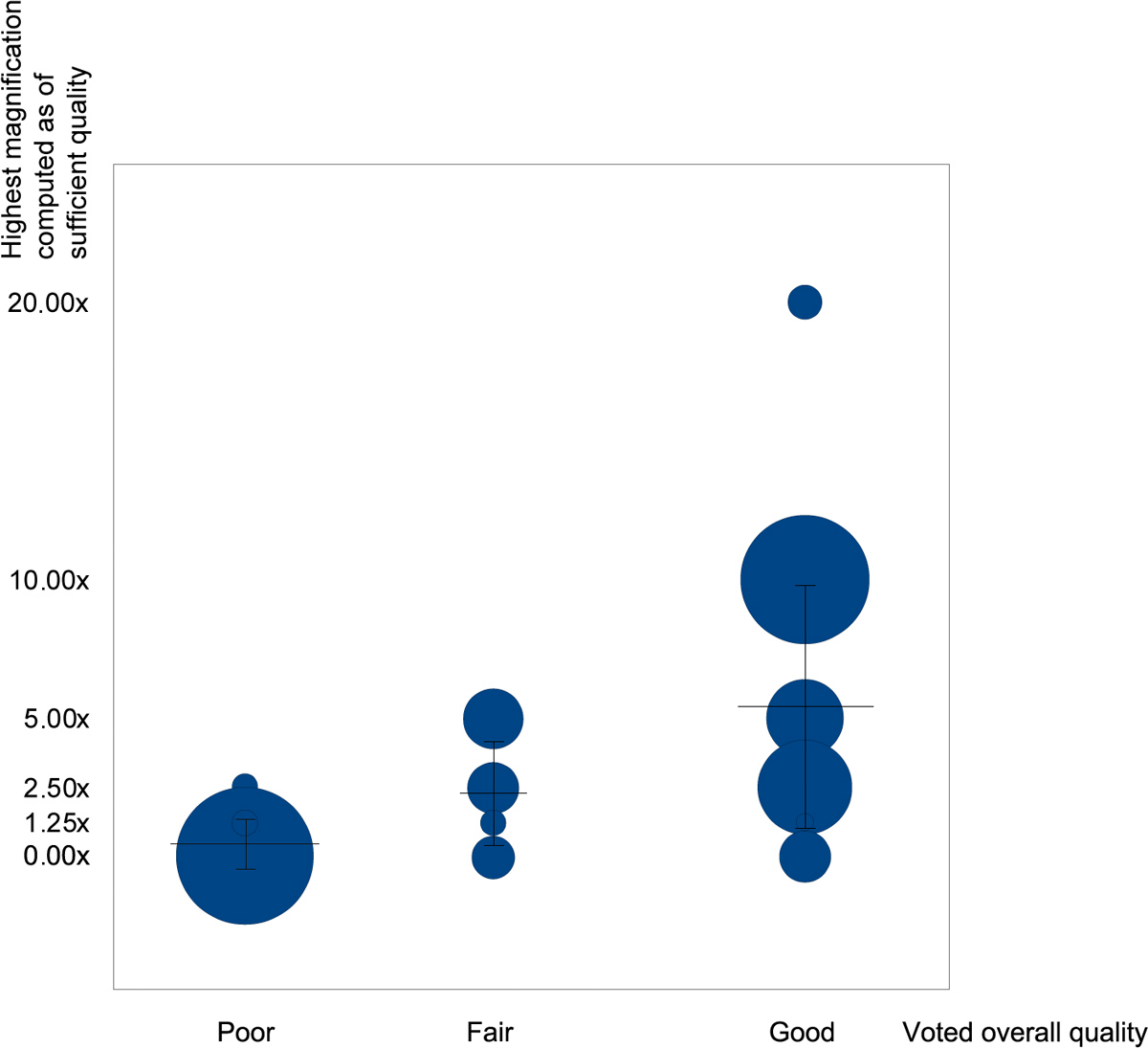
**Comparison between voted overall quality and best detected magnification**. Distribution of the (human assessment; computer assessment) pairs for 100 WSI with various blurred levels. Human assessment is distributed in three categories: poor/fair/good quality for diagnosis. Computer assessment is distributed in five different magnifications (from 1.25× to 20×): it shows the highest acceptable magnification for a WSI, i.e. the magnification for which the WSI computed quality is sufficient, implying that higher magnifications of this WSI are of insufficient quality. The surface of the disk is proportional to the number of identical pairs. The horizontal bars represent the mean of the highest acceptable magnifications of the computer assessment at each category of human assessment, with vertical bars as their respective standard deviation.

For the web survey, the results [13] obtained after the visual analysis on 100 WSI by 22 pathologists are shown in Figure 2. The results found by our algorithms are fully consistent with the pathologists' answers to the survey: the mean computer assessment is 1.25× with a standard deviation of 2.37× in the "poor" human assessment category, increasing to 2.90× with a standard deviation of 2.51× in the "fair" category and to 6.35× with a standard deviation of 5.57× in the "good" category.

However, the survey showed that the human assessment do not entirely correspond to the computer assessment, due to the fact that some diagnoses do not need high magnification for human eyes to be done. Indeed, a high computer quality at low magnification was sometimes enough to give a correct diagnosis (blue disks on the lower right part of Figure 2), but a high-level computer assessment (computed high quality at high magnification) always corresponded to a high level human assessment (blue disks on the upper right part of Figure 2).

As further improvements of our method, we will contextualize the assessment by refining the thresholds depending on staining and lesion.

**Figure 3:**
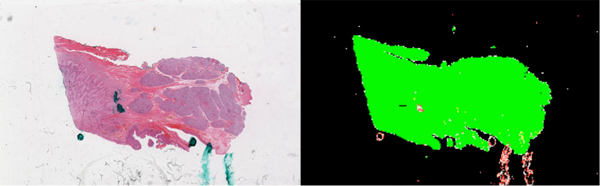
**WSI and sharpness result**. A 3.2 billion pixels Whole Slide Image extracted as an array of images of and its sharpness map, analyzed at a 3-billion-pixel-per-minute rate. The analysis has detected regions of interest in 29.4% of the slide. Among these, 92.9% are sharp (in green), 1.7% are partially sharp, and 5.1% are blurred.

In terms of computing speed, Zerbe et al. [5] showed a distributed computing model to assess the focus sharpness of a WSI, generating a focus assessment map of the WSI at a given magnification level in around 6 minutes per gigapixel per computer. We analyzed on our testing server 8 complete 1.73 gigapixel digital slides in 400 seconds as eight distinct threads, equivalent to 34 Megapixels per second or 2 gigapixels per minute, per computer. Already 12 times faster than the previous method, we are currently optimizing the program into a multi-thread, multi-node parallel processing system using C++ with OpenMP and OpenMPI libraries to scale it up to match demanding industry requirements. The WSI sharpness analysis Java library we designed is a Service Provider Interface (SPI): an Application Programming Interface (API) aimed at being extendable by third parties. The full library (JAR file) weighs 12 KB and is fully operational for sharpness analysis of single images (tiles), and for array of images such as the WSI in Figure 3.

The speed of analysis is in average 3 billion pixels by minute using our development environment with the JAI (Java Advanced Imaging) API.

The Python mono-threaded interface was tested with an average rate of 1 billion pixels by minute.

We designed 4 sharpness assessment programs based on our Java multithreaded library:

One java program using any regular image file (JPEG, PNG, TIFF, GIF, BMP...) or array of image files, and returning a list of values as described in our paper, with text-only results.

**Figure 4:**
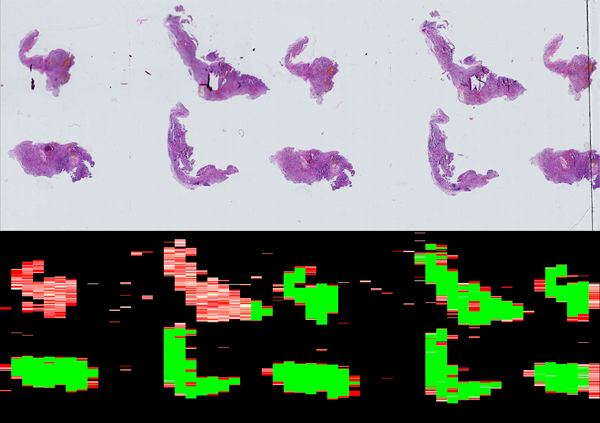
**NDPI sharpness results**. Hamamatsu NDPI 18.7 billion pixel Whole Slide Image and its sharpness map, analyzed at a 3-billion-pixel-per-minute rate. The analysis has detected regions of interest in 22.0% of the slide. Among these, 61.3% are sharp (in green), and 38.73% are blurred.

One java program using WSI in the Hamamatsu NDPI file format, and returning global results for the slide sharpness at each magnification, as well as a sharpness map of the WSI summarizing the results with colors relative to the sharpness assessed (green for sharp regions, yellow for partially sharp regions, red for blurred regions). Implementation is shown in Figure 4.

One java program using JPEG files structured as required by the Google Maps format: a tree structure containing folders numbered as such (starting with 0 and incrementing as required): Magnification-index/Y-position/X-position.jpg and returning similar results (text and image, as described above).

**Figure 5:**
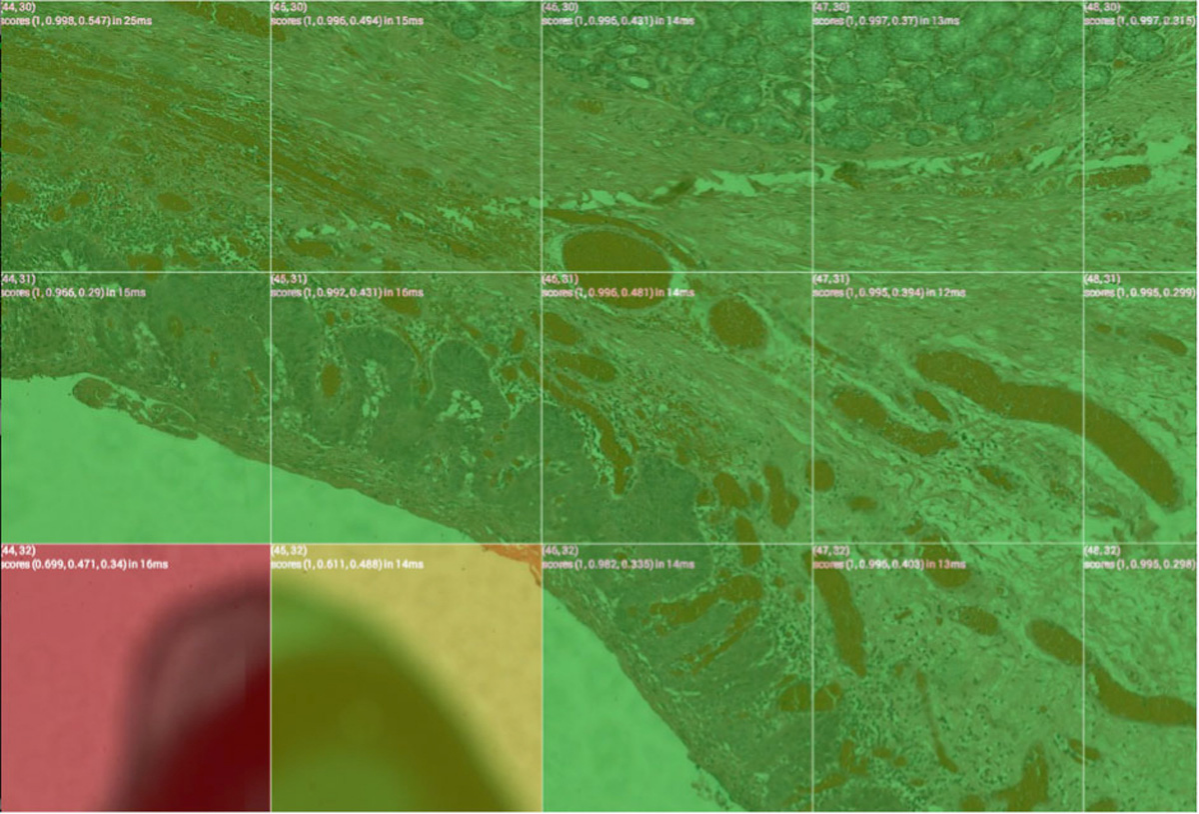
**WSI in the Google Maps format**. Aperio SVS Whole Slide Image converted to the Google Map format, viewed and analyzed in real-time using NYUVM's web-viewer plugged in with our Java sharp-ness analysis multithreaded library.

One web application using JPEG files structured as required by the Google Maps format, to be viewed with the NYUVM. We connected our Java library to NYUVM by adding Ajax functions, triggering socket connections with PHP to receive a JSON array containing the results of the sharpness analysis for each visible tile, and display the sharpness results of each tile in real time. The sharpness analysis of the tiles are computed and sent concurrently and faster than the images are displayed, with no slow-down compared to the original NYUVM viewer, thereby in real-time [17]. Implementation is shown in Figure 5.

Programs 1., 3. and 4. also have Python implementations.

Our Python implementations were 3-times slower in average than our Java implementations as we haven't yet used Python's multithreading capabilities. We are also currently developing multithreaded Python and Open MPI C++ implementation.

Tests were made on 5000 single images, 200 WSI in Hamamatsu formats, 100 WSI in Aperio SVS format converted to the Google Maps format.

It is currently being implemented in the French national project FlexMIm and additional results should be provided in the last quarter of 2014.

In this perspective, we think that integrating these programs in the WSI acquisition systems can tremendously increase the quality of each scanned WSI without significantly slowing down the acquisition workflow. It will also most definitely speed up the quality assurance process, currently done manually after the WSI has been acquired, and by a subjective visual-only assessment. Implementing these libraries, coupled with regions-of-interest detection algorithms, may enhance intelligent image transfer protocols by sending and displaying the WSI's regions marked as being of interest and of highest quality before other regions. On another matter, image compression algorithms could be designed to favor sharp regions, by requiring lossless methods, and, on the contrary, accept lossy methods to be used on blurred regions. Should such quality assessment scores become part of the WSI's metadata, they may help standardize image quality requirements for digital pathology.

## Conclusions

As quality assurance is crucial in a context of daily use in diagnostic pathology, we have developed a fast and reliable no-reference quality assessment library for WSI and digital images in general.

The proof of concept for this no-reference and high-speed quality assessment tool for virtual slide was developed in 2010, thoroughly tested and described in 2012.

Development of Service Providing Interfaces and Application Programming Interfaces has been carried out in 2012-2014, and implementation started in French national projects in 2013.

Applications based on these libraries can be used upstream, as calibration and quality control tool for the WSI acquisition systems, or as tools to reacquire tiles while the WSI is being scanned. They can also be used downstream to reacquire the whole slides that are below the quality threshold for surgical pathology analysis.

We think that implementing these libraries could be used as an intelligent accelerator to viewing WSI by sending and displaying the regions marked as being of highest quality before other regions.

Such quality assessment scores could be integrated as WSI's metadata shared in clinical, research or teaching contexts, for a more efficient medical informatics workflow.

## List of abbreviations

WSI: Whole Slide Images; NYUVM: New York University's Virtual Microscope; JAI: Java Advanced Imaging.

## Competing interests

### Financial competing interests

David Ameisen is a recipient of a postdoctoral fellowship grant from the FlexMIm project (2013-2014), and was a recipient of a doctoral fellowship grant from Aurora Interactive (2008-2011); Olympus provided him travel reimbursements for one scientific meeting presentation in 2008. None of these organizations are financing this manuscript.

David Ameisen and Philippe Bertheau have published one patent (WO2012080643A1) relating to the content of this manuscript. They are receiving salaries from Université Paris Diderot that has applied for this patent. SATT idfinnov is currently funding this work (2014).

No other author has financial competing interests.

### Non-financial competing interests

None

## Authors' contributions

DA participated in the design of the study, the development of the libraries, and drafted the manuscript, CD carried out the Hamamatsu tiles extraction and participated in the design of the study, VP participated in the design of the study, FB participated in the statistical analysis, MB participated in the statistical analysis, LL participated in the statistical analysis, AJ participated in the design of the study, PB participated in the design of the study, and drafted the manuscript, JBY participated in the design of the study, the development of the libraries, and drafted the manuscript. PB and JBY have contributed equally to the work. All authors read and approved the final manuscript.
